# A Discrete Model to Study Reaction-Diffusion-Mechanics Systems

**DOI:** 10.1371/journal.pone.0021934

**Published:** 2011-07-11

**Authors:** Louis D. Weise, Martyn P. Nash, Alexander V. Panfilov

**Affiliations:** 1 Department of Theoretical Biology, Utrecht University, Utrecht, The Netherlands; 2 Auckland Bioengineering Institute and Department of Engineering Science, The University of Auckland, Auckland, New Zealand; 3 Department of Physics and Astronomy, Ghent University, Ghent, Belgium; Queensland Institute of Medical Research, Australia

## Abstract

This article introduces a discrete reaction-diffusion-mechanics (dRDM) model to study the effects of deformation on reaction-diffusion (RD) processes. The dRDM framework employs a FitzHugh-Nagumo type RD model coupled to a mass-lattice model, that undergoes finite deformations. The dRDM model describes a material whose elastic properties are described by a generalized Hooke's law for finite deformations (Seth material). Numerically, the dRDM approach combines a finite difference approach for the RD equations with a Verlet integration scheme for the equations of the mass-lattice system. Using this framework results were reproduced on self-organized pacemaking activity that have been previously found with a continuous RD mechanics model. Mechanisms that determine the period of pacemakers and its dependency on the medium size are identified. Finally it is shown how the drift direction of pacemakers in RDM systems is related to the spatial distribution of deformation and curvature effects.

## Introduction

Reaction-diffusion (RD) partial differential equations describe important spatio-temporal phenomena, including waves and patterns in a variety of chemical, physical, and biological systems. Important examples of these phenomena include waves in the Belousov-Zhabotinsky (BZ) reactions [1], [2], waves of CO oxidation on platinum surfaces [3], waves of spreading depression in nerve tissue [4], and the morphogenesis of *Dictyostelium discoideum* (Dd) [5], [6]. In the heart, electrical waves of excitation propagate through the tissue and initiate its contraction. RD-equations have been successfully applied to model normal and abnormal wave propagation in cardiac tissue, such as rotating spiral waves, whose initiation may result in life-threatening arrhythmias [2], [7]. In many of the systems mentioned above, wave propagation is accompanied by a deformation of the medium. Important examples include the chemotactical motion of cells during Dd-morphogenesis [6], the swelling and deswelling of a polymeric gel in the BZ reaction [8] and the contraction of the cardiac muscle [9]. As the heart contracts, its deformations feed back on the process of wave propagation. This important phenomenon, called mechano-electrical feedback, has been extensively studied in cardiac electrophysiology [10].

To model the effects of deformation on wave propagation in RD systems, it is necessary to describe the underlying mechanical phenomena in terms of the RD process. As such, a coupled reaction-diffusion-mechanics (RDM) framework was introduced in [11] and applied to study cardiac tissue. In particular, the RD equations were combined with the equations of finite deformation continuum mechanics. With this approach several important effects of deformation on wave propagation were identified such as self-organized pacemakers, spiral wave drift, and break-up of spiral waves [12], [13].

Continuum mechanics is among the most valuable and widely used approaches in engineering and modeling studies, however, it does not explicitly describe the particular micro-organization of a medium, which might be important for certain aspects of RDM systems. Cardiac tissue, for example, consists of individual cells that form layers of muscle fibers, which are tightly packed and organized by an extra-cellular matrix into branching sheet structures [14], [15]. To study how this affects the elastic properties of the heart, discrete models with similar micro-structure need to be developed. Discrete models are computationally efficient and widely used in various applications such as computer graphics [16], medical tissue visualization [17], and the development of elasto-mechanical models of anisotropic materials [18] such as heart tissue [19], [20]. Discrete models are also used to describe discontinuous deformations in the case of fracture, plastic deformation, and mass mixing processes [21], [22].

In this paper, discrete elastic modeling is coupled with FitzHugh-Nagumo type RD partial differential equations to study RDM systems. First, the process of the setting up of the discrete RDM (dRDM) model is described in detail, and computational and numerical aspects are addressed to discuss the macroscopic elastic properties of the medium. Secondly, as an illustration of the new modeling approach, the dRDM model is applied to study pacemaking activity in the RDM system shown in [12]. This illustration demonstrates that the dRDM model adequately reproduces all of the important phenomena of pacemaker dynamics previously found with a continuous RDM model [12]. The computational efficiency of the dRDM approach allows more detailed investigations into the mechanisms determining important properties of the pacemaking activity. Next, the factors determining the period and drift of the pacemakers found in [12] were identified. Overall, the value of the dRDM modeling approach, as a tool to study RDM systems, is demonstrated.

## Methods

### Reaction diffusion model for cardiac excitation

In this paper, as in [12], the Aliev-Panfilov model [23] for cardiac excitation is used as the RD part of the dRDM model. Of course the same approach can easily be applied to any RD model describing cardiac excitation or any other reaction-diffusion process. The purpose of using a cardiac RD model is to reproduce the time course of the transmembrane potential. The transmembrane potential changes due to ionic currents, which flow through voltage-gated ion channels of the cardiac cell membrane. The reaction part of the model describes these currents either in a general form (in low dimensional models) or on the basis of detailed experimental data (ionic models). The spatial coupling between cells in the RD approach is demonstrated by the Laplacian operator [24]. The Aliev-Panfilov model [23] provides a low-dimensional description of excitation for cardiac cells. The equations of this model are
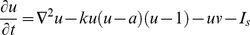
(1)

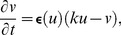
(2)where 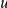 and 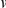 are normalized representations of the transmembrane potential and the conductance of a slow repolarizing current, respectively. These variables are quantified in this paper in dimensionless units, for 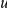 excitation units [e.u.], and for 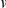 recovery units [r.u.] are used. The term 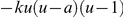 in Eq.1 describes the fast excitation process of the AP. The parameter 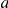 represents the threshold of activation and parameter 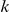 controls the magnitude of the transmembrane current. In this study, 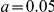 and 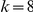 were used in all computations. 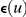 is a step function setting the time scale of the recovery and the contraction process: we set 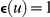 for 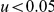, and 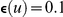 for 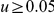 (also used in Eq.3). The term 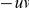 describes the repolarizing current of the recovery process. The term 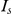 is the stretch activated depolarizing current described in Eq.9. In a non-deforming medium Eqs.1,2 with these parameter values describe non-oscillatory cardiac tissue providing stable propagation of excitation waves.

### Excitation-contraction coupling model

Following [12] the excitation-contraction coupling is modeled using
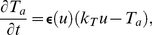
(3)where 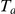 modulates the active contraction force in Eq.5 to associated mass points of the medium. The parameter 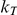 controls the amplitude of the contraction twitch, where 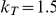 was used in all simulations. The identical term Eq.3 was used in the continuous RDM modeling approach of Panfilov et al. [12] to account for the active stress. The model formalism of Panfilov et al. from [12] is an important benchmark for the dRDM model in this paper, and is referred to in the following text as the PKN description.

### Mass-lattice model

**Figure 1:**
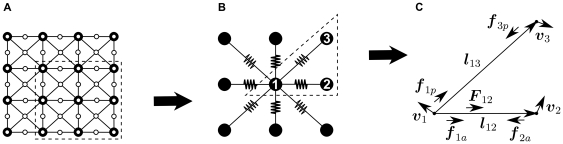
Coupled mechanical and RD mesh. **(A)** Coupled mechanical and RD mesh. The mass points are indicated as large black dots. The finite difference points to solve Eqs.(1)-(3),(9) are indicated as small white dots. The lattice springs are indicated as black lines. **(B)** Unit cell of the 2D lattice. Mass point **1** and its four horizontal and vertical nearest neighbors and four diagonal next-nearest neighbors are connected with direct active and diagonal passive springs. Lattice springs are indicated by zigzagging lines (fat lines for active and thin lines for passive springs). Dotted contours indicate insets for the associated subfigures. **(C)** Vectors used in Eqs.(4)–(6) for calculating lattice interactions.



To model the mechanical properties in the dRDM approach a 2D lattice that consists of material points connected by springs (Figure 1A) is used. The unit cell of this 2D lattice is shown in Figure 1B. Each mass point is connected to its four nearest neighbours in horizontal and vertical directions at resting distance 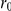 and to its four next-nearest diagonal neighbors at resting length 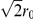. All springs follow Hooke's force-displacement relation and horizontal and vertical springs may produce additional active contraction forces. Following the continuous PKN approach [11], elastostatics is assumed in this dRDM model, i.e. the stationary deformations corresponding to each given configuration of active forces and boundary conditions are computed. The procedure is outlined as follows. At steady state, the total force at each node is zero. If the configuration of the active forces is changed, the force balance at the mass points will be violated which results in the motion of the mass points to a new stationary configuration. For efficient computations of this system, viscous forces were added to dampen possible oscillations. The formal formulation of the approach is given below: Figure 1C demonstrates main forces and the displacements of active and passive lattice springs connecting the mass point **1** to the mass points **2** and **3**. The positions of the mass points are given by 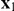, 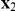, 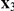, with the corresponding velocities 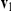, 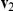 and 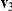. Mass points **1** and **2** are connected by an active spring. The force generated by this spring on the mass points is given by

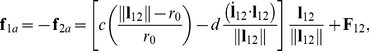
(4)where 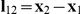 is a vector along an active spring, 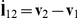 is the time derivative of the spring vector 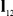, parameters 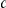 and 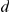 are the stiffness and ‘damping’ constants, respectively (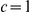 in all simulations), and 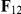 is the active force between mass points **1** and **2** given by
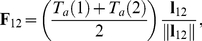
(5)where 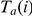 is the value of variable 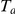 from Eq.3 at mass point 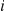. Mass points **1** and **3** are connected by a passive spring. The force generated by this spring is given by
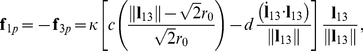
(6)where 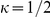 is the stiffness ratio between active and passive springs. It is assumed that each node here is subject to Newton's law of motion:
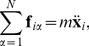
(7)where 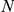 is the number of springs connected to mass point 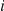, 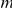 is the mass of a point, and 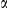 indicates connected springs. By solving Eq.7 to mechanical equilibrium (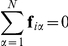), the steady state configuration of the lattice for each given distribution of active forces generated by the RD process is found. Note that the ‘mechanical’ time variable 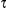, as well as the parameters viscosity 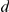 and the mass of a node 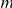, have no physical relevance in this model, but each fulfill a pure numerical role. The mass 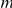 of each mass point was set to 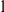 (dimensionless mass units).

#### Material properties

The elastic mechanical properties of the dRDM model are determined by the geometry of the lattice unit cell and the stiffness of the springs. In classical continuum mechanics, elastic properties are represented by constitutive relations between the corresponding stress and strain tensors. Constitutive relations are successfully used to describe the elastic properties of many materials including biological tissues. It is possible to formulate the elastic properties of this paper's mass-lattice model in terms of an equivalent continuous material. The mass-lattice model (Figure 1B) in this paper was extensively studied for various aspects of elasticity. In most cases the mass-lattice model was studied under conditions of small deformations (linear elasticity). The relation between stress and small strain of this mass-lattice model was shown to be expressible in the form of a 4th rank elasticity tensor 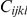, and its coefficients can be directly derived from the spring constants of the system [25]. Furthermore, it was demonstrated by Schargott et al., that if the stiffness ratio is 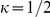, the lattice is macroscopically isotropic [26]. This implies that the linear elasticity tensor is rotationally invariant. In this case the constitutive relations simplify to the generalized Hooke's law [26]

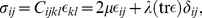
(8)where 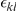 are elements of the small-strain tensor 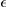, 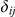 is the Kronecker delta and 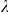 and 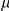 are the Lamé coefficients, which in this case are equal to each other [26]. The elastic coefficients of this material are Young's modulus 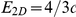 (where 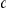 is the spring stiffness as defined above), and Poisson's ratio 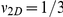 [26]. An extension of this material relation for finite deformations can be found in [27]. Krivtsov et al. explained in [27], that even for non-linear deformations, the elastic properties of the mass-lattice model used in this paper can be approximated by a generalization of Hooke's law for finite deformations (Seth material) [28]. The Seth material relation is similar to Eq.8 but uses the Almansi strain tensor for finite deformations instead of the small strain tensor 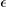. Therefore, for 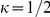 used in the simulations of this paper, the mass-lattice model is applicable for the description of an isotropic material undergoing non-linear deformations.

**Figure 2:**
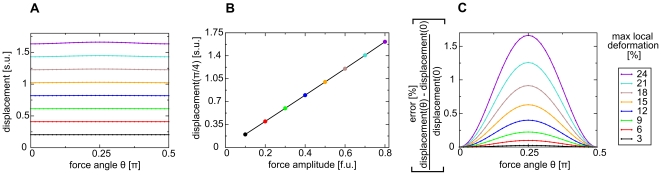
Seth material relation. A force was applied at the center point for different amplitudes and 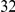 orientations 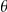 between 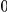 and 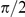. An angle of 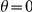 corresponds to a force perpendicular to the border. **(A)** Isotropy: displacement is approximately constant for different force angles 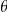 over a range of force amplitudes. **(B)** Displacement of the center point *vs* force amplitude (at 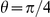). **(C)** Error of displacement (relative to displacement at 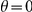) *vs* force angle 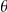. For these simulations a system size of 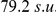 and 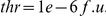 were applied.

Numerical studies were performed to illustrate the material properties for conditions used in this paper's simulations. A deformation field was created by applying a force at the central mass point of an extended 2D lattice, whose boundaries were fixed in space (isometric boundary conditions), leading to finite displacements and local deformations. Next, Eqs.4–7 were solved to mechanical equilibrium and the displacement of the mass point from its initial position and the angle between the force and the displacement vector were calculated. Similar computations were performed for different orientations and amplitudes of the force vector. This comparison (Figure 2A) demonstrates that the 2D mass lattice model used in this paper can be considered as a good approximation to a macroscopically isotropic material, at least for local deformations of up to 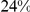. The displacement error for 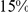 local deformations was 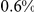, and for 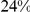 local deformations 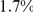 (Figure 2C). The angle deviation of the displacement vector and the applied force was 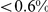 for maximal local deformations up to 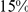 (data not shown). Furthermore, Figures 2A and 2B demonstrate, that a linear relationship between force and maximal local deformation, and between force and the displacement of the central mass point holds true for the whole range of studied force amplitudes, i.e. up to maximal studied local deformation of 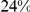. In summary, the mass-lattice model described here with 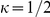 describes an isotropic medium, which follows a linear force-displacement relation that can be approximated by the Seth material relation.

### Electromechanical feedback

The deformation of cardiac tissue alters the process of wave propagation. It has been shown in studies of excised cardiac tissue and the whole heart that the direct physiological influence of contraction on cardiac tissue is given by a depolarising stretch-activated current 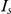 through stretch activated channels [10]. Experimental studies have shown, that these channels are activated instantaneously with mechanical stretch and follow a linear current-voltage relationship [29], [30]. Linear models have been proposed for 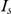 [31], [32], and have also been applied in other electromechanical models [12], [13]. Following these previous studies the equation
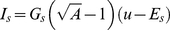
(9) is used where 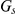 and 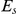 are the maximal conductance and reversal potential of the stretch activated channels. Following [12], [13], 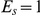 was chosen. 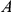 is the surface area of a quadrilateral formed by 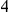 neighboring mass points (see Figure 1A) normalized using the reference surface area of this quadrilateral in undeformed state (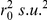). The stretch activated current is active only if 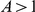 (stretch). The value of 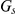 is a main determinant of the effect of 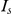. The value 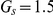 was used for all computations in this paper unless stated otherwise.

### Numerical methods

The dRDM model was solved using a hybrid approach, combining an explicit Euler scheme for the RD Eqs.1–3,9 with a Verlet integration scheme [33] to solve the equations for the motion of the mass points Eqs.4–7. The position of a mass point 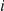 for the integration time 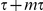 is computed by
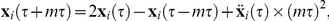
where 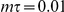 is the Verlet integration time step and 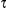 is the integration time. For the very first computation
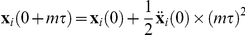
was used. The acceleration of a mass point 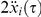 is given by Eq.7. At each time step the velocities of the mass points are calculated by
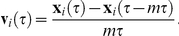



The solution procedure of the dRDM model is as follows: after 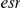 time integration steps for the RD model and electromechanical feedback (Eqs.1–3,9), the equations of the mechanical model (Eqs.4–7) are solved for all of the springs until the sum of forces for each mass point is under the convergence threshold 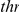 (dimensionless force units [f.u.]). Euler computations were performed on a quadratic deforming grid of finite difference points using no-flux boundary conditions. For all simulations, an Euler integration time step of 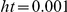 (dimensionless time units [t.u.]) and a space integration step of 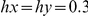 (dimensionless space units [s.u.]) were used. Each surface area element 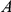 consists of 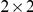 directly neighboring mass points and 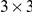 electrical grid points (Figure 1A). For grid points at the boundary of two (or more) surface area elements 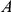, the average value of these normalized surface areas was used to compute 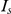 (Eq.9). When solving the mechanical equations Eqs.4–7, the boundaries of the medium were fixed in space. This approach is commonly applied in computational studies on cardiac physiology [12], [13]. It corresponds to the isometric contraction in tissue experiments, and is similar to the isovolumic phases of the cardiac cycle at the whole organ level.

### Model validation

For the integration of the dRDM model Eqs.1–7, 9 several parameters of the numerical scheme were chosen. This section demonstrates how parameters were set to assure accurate and efficient computations.

#### RD integration parameters

The RD Eqs.1–3 were solved using the finite difference approach with an explicit Euler integration scheme. Previous studies of the Aliev-Panfilov RD model used space steps of 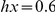 [12] to 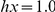 [34]. To assure high spatial resolution of the dRDM model, a space step of 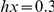 was used in this study. A time step of 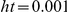 was applied to assure efficient computation of the coupled mechanical model Eqs.4–7 (see following section ‘Electrical and mechanical grids’).

#### Damping-stiffness-ratio and Verlet integration time step

The equations describing the motion of the mass points Eqs.4–7 represent a system of coupled, driven, damped oscillators. As elastostatics was assumed in this work, the damping-stiffness ratio 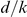 in this case is just a numerical parameter that controls the rate of convergence of the lattice mass points to their equilibrium positions. It seems logical to use the largest possible value of 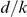 to assure the fastest possible convergence. However, large values of 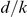 lead to numerical instabilities, because of the counter-play of the elastic and viscous forces. It was found that the use of 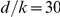 resulted in fast convergence of Eq.7, with no numerical instabilities and convergent results, so this ratio was used for all simulations in this paper. For solving the mechanical equations Eqs.4–7 an integration time step of 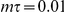 was used, which was found as the maximal time step that allowed fast stable convergence of the lattice mass points to their new configuration.

#### Electrical and mechanical grids

**Figure 3:**
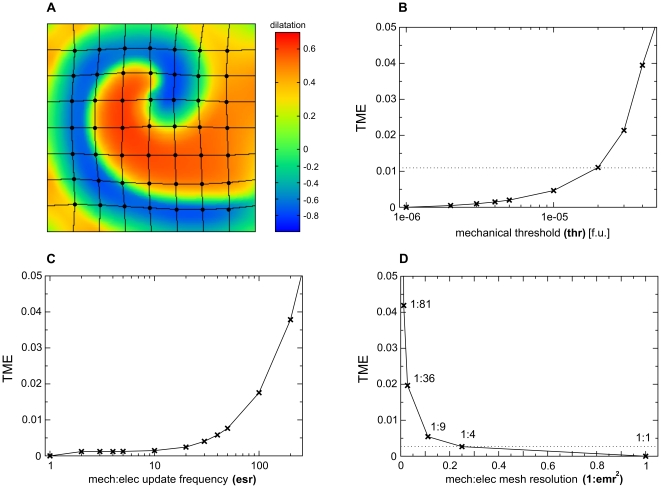
Determining mesh parameters. **(A)** Experimental setup: trajectories of 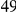 mass points (large big dots) are traced during a spiral wave rotation during 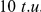 in a medium size of 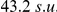 along each side. The color spectrum indicates (local) dilatation (scaled) in the medium (
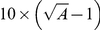
). Maximal local deformations 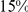. **(B)** 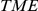 *vs* 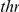 with 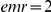, 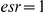 and using 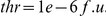 to compute reference trajectories. The dotted horizontal line indicates the parameter chosen for further computations (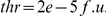). **(C)** 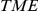 *vs.* 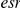 with 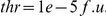, 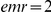, and 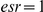 to compute reference trajectories. **(D)** 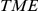 *vs.* 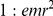 with 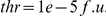, 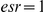 and 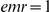 to compute reference trajectories.



In RDM systems, the deformation is more smoothly distributed in space compared to the state variables of the RD equations [11]. It is therefore possible to use a coarser grid for the solution of the elastostatics equations compared to the integration of the RD equations. Furthermore, the slower mechanical dynamics enabled the update of the mechanical configuration after a number of RD integration steps had been performed [11]. To appropriately choose the parameters that define the relation between the mechanical and RD grids, one must understand how they affect the accuracy of the simulations. These parameters include: the ‘mechanical update rate’ 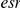 (number of RD integration steps after which the new mechanical configuration is computed), and the ‘spatial resolution’ of the mechanical grid (the relative resolution of the mechanical and RD grids). The spatial resolution is expressed as the ratio of the total number of mechanical nodes to the total number of RD nodes. This means, that if the mechanical grid is twice (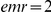) as coarse as the RD grid, then the ratio of total numbers of nodes will be 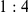 (in general 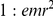 in 2D). Additionally, the accuracy of the solution of equation Eq.7 also affects the mesh coupling and the accuracy of the dRDM model. The accuracy for the solution of Eq.7 is characterized via a threshold parameter 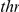, which determines the convergence of the system to elastostatics (the sum of forces at each mass point must be smaller than 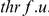). In order to determine how these parameters affect the accuracy of the dRDM model, the following numerical experiment was performed: First, deformation patterns that occurred during the stable rotation of a spiral wave for the duration of 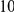 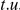 (which is 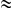 1/3 period) were selected as the reference solution. These reference patterns were calculated with the integration parameters listed in the caption of Figure 3. Next, an investigation of how less accurate parameter settings affect the accuracy of the solutions was performed. This procedure was applied for various parameter sets and the trajectories of 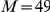 mass points equally distributed in the medium were traced (Figure 3A). The deviation of these trajectories was computed from the reference solution for each simulation time step 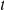. To estimate the mean absolute error of the mass points for a time step 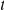 an ‘instantaneous’ error norm 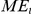 was defined as

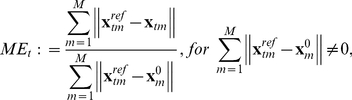
(10)where 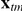 is the position of mass point 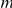 at time step 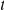 for a given solution, 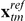 is the corresponding position of this mass point for the reference solution, and 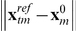 is the displacement of the point 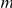 from its initial position for the reference solution, and 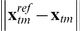 is the difference between the given and the reference solution for point 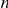 at time step 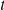. To estimate the total mean error, an error norm 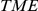 for the whole experiment over 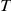 time steps was defined as
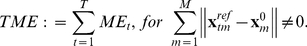
(11)


Note that for testing patterns used here, the instantaneous error norm 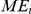 did not vary significantly during the studied time period (data not shown). Therefore, it is adequate to use 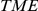 to approximate the total mean error. In Figure 3B the effect of 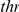 on 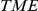 is shown. For 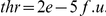 the estimated total mean error due to this parameter variation was about 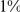. Figure 3C shows the influence of the mechanical update rate 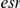 on the accuracy of the model. As a result, the total mean error due to this parameter is expected to be less than 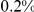 when 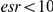. However, for this study we chose to fix 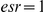, because of the observation that the more frequent update of the mechanical grid (together with a small time step to integrate the RD equations) resulted in significantly faster convergence of the iteration procedure and hence decreased the overall simulation time (data not shown). Figure 3D shows the effect of the relative spatial resolution of the mechanical and electrical grids on 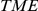. For 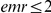, the 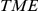 is around 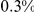. Based on that the parameters were chosen to: 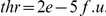, 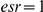 and 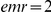. In summary, this parameter choice is expected to cause a total mean error of around 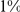 mainly due to the choice of the mechanical threshold 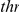.

### Computational performance

This section analyses and compares the computational performance of the dRDM and PKN approaches. In particular, the scaling of the computational times for the two modeling schemes versus the number of mechanical nodes 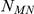 was investigated using the following simulations. A radially spreading wave was initiated at the center of an excitable medium using a point stimulus and the subsequent activity was simulated for a duration of 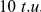 using serial processing on a personal computer with a 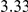
GHz Intel Xeon X5680 processor. To compare the computational scaling, we used identical mechanical and electrical grids for both models, with 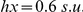 and 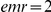. Even though a direct comparison of the nodal resolutions of both modeling approaches (mass points for dRDM and finite elements for PKN) is difficult in terms of mechanical accuracy, it is important to note that the calculation of stretch activated current 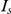 (Eq.(9)) is directly affected by he resolution of the mechanical nodes.

**Figure 4:**
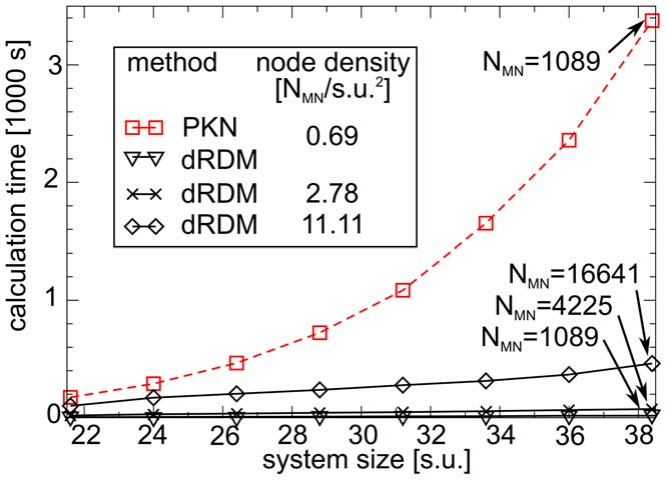
Comparison of computational performance of dRDM and PKN. Calculation time is plotted against medium size. A radially spreading wave was initiated at the center of the medium of PKN and dRDM models and simulated for a duration of 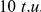. Parameters for PKN were as in [12], but 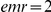 was used to achieve same mechanical node density as the dRDM with 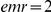 and 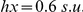 (
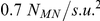
). Models with increased mechanical node densities were also analysed using the dRDM approach with 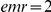, 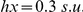 (
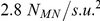
), and 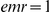 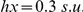 (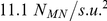).

Figure 4 illustrates computation time 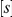 plotted against the system size for the different approaches. For the PKN model, the computation time increased non-linearly with the number of finite element mesh nodes. This was primarily due to the N-squared scaling for the solution of the linearised equations, whilst the element stiffness calculations scaled approximately linearly (data not shown). On the other hand, for the dRDM models the total CPU-time increased approximately linearly with the number of lattice mass points for the system sizes considered here (linear regression analyses showed 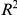 values better than 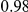). Furthermore, the positions of the mechanical nodes for the dRDM simulations (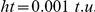, 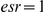) were updated 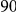 times more frequently than the nodes of the PKN model (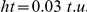, 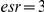). Despite this, the dRDM model with 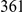 mechanical nodes (system size 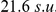) computed the results 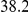 times faster than the PKN model with the same nodal resolution, whilst the dRDM model with 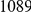 mechanical nodes (system size 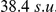) solved 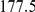 times faster than the PKN approach. Figure 4 also illustrates that the resolution the dRDM model can be substantially increased (here shown up to 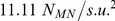 with 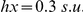 and 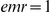), whilst its computational performance allows such computations for larger RDM systems.

Figure 4 compares the computational performance of PKN and dRDM simulations with a mechanical node density of 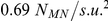 (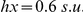 and 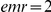). For the main results simulations presented in this paper, a 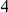 times higher mechanical node density 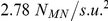 (dRDM 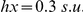 and 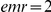) was used with the dRDM model. Compared to these dRDM simulations previous PKN research used simulations with up to 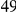 times lower node density (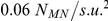 with 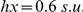 and 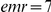) [12]. If we compare the computational performance for a typical simulation in this paper (e.g. large system in section ‘Mechanisms of pacemaker drift’, which contains 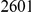 mechanical nodes with a medium size of 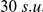), then we estimate that the dRDM approach will be 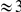 magnitudes faster than the PKN model (PKN requires 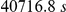, and dRDM requires 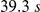 for the upper experiment with that number of mechanical nodes). Thus, the application of the PKN model for higher resolutions and larger system sizes is not computationally tractable for studying extended duration model simulations. It is possible to use advanced numerical techniques to improve the numerical performance of finite element methods such as the PKN approach, however that is beyond the scope of this study. The primary aim of this study was to develop a simple and efficient alternative to the PKN approach for the study of basic effects of deformation on wave propagation in excitable media. The dRDM approach provides a computationally tractable method for studying large RDM systems with high temporal and spatial numerical resolutions. The usefulness of the dRDM approach is illustrated in the following results section.

## Results

We have introduced a discrete modeling framework to study the basic properties of RDM systems. We first show that the dRDM approach is able to reproduce some previously reported results on pacemaking activity, which were identified using the PKN model [12]. The RD model in [12] is identical to Eqs.1–3 in this paper. In addition, no flux boundary conditions for the RD equations and fixed boundaries of the mechanical mesh were used in the present study, as reported in [12]. On the other hand, [12] uses a continuum mechanics formulation that follows the Mooney-Rivlin material relation. The Mooney-Rivlin relation shares similarities with the Seth material relation used in this dRDM model, because both constitutive relations describe isotropic elastic mechanical response. However, the Mooney-Rivlin material relation describes a nonlinear force-displacement relationship for finite deformations. Therefore, we did not seek an exact correspondence of the two approaches, but rather a qualitative agreement as a reflection of the underlying basic mechanisms determining pacemaker dynamics.

### Pacemaker drift

In [12], Panfilov et al. reported on the phenomenon of automatic pacemaking activity in coupled RDM systems. The main objective of this section is to test if the dRDM approach reproduces important mechanisms on self-organized pacemakers that were identified with the continuous PKN modeling framework [12].

To begin, the phenomenon of self-organised pacemaking activity is described. It has been found that a single electrical or mechanical point stimulus can cause the formation of a pacemaker in a RDM medium with non-oscillating RD kinetics. Pacemaking activity occurs because the contraction of the medium that follows a radially propagating wave of excitation subsequently stretches the medium in the neighborhood of the initiation site. This stretch induces a depolarizing stretch activated current 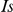 (Eq.9) that initiates a subsequent excitation wave. The location of this pacemaker may drift over the course of time depending on the position of the initial stimulus [12]. Two main drift directions were identified: to the center of the medium (for larger medium sizes) and to the boundary (for smaller medium sizes) with an intermediate regime involving multiple symmetric attracting points.

**Figure 5:**
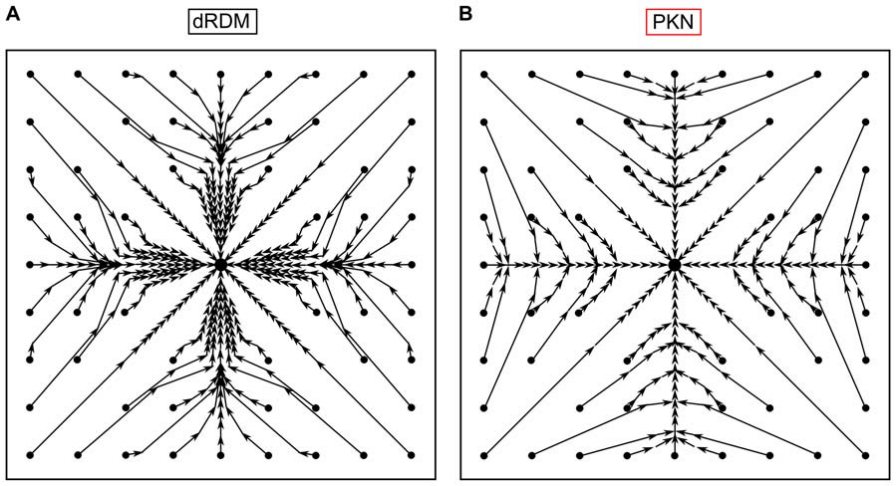
Pacemaker drift in large medium (size 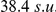). Small black dots indicate positions of point stimuli and the arrows indicate drift directions and the estimated positions of sequential action potentials (slow drift is indicated by short arrows). Attractors are indicated as big black dots. **(A)** dRDM model with 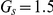 **(B)** PKN model with 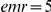, other parameters as in [12].

Simulations with the dRDM and the PKN models were performed, which showed that all effects found with the PKN model [12] were qualitatively reproduced by the dRDM approach. In particular, the dRDM model reproduced the phenomena of pacemaking activity as well as the dependence of pacemaker drift on the location of the initial stimulus and the size of the medium. Figure 5A shows typical drift patterns for a large dRDM model. The pacemaker drifted to the center of the medium from all initialization locations. Figure 5B shows the same experiment performed with the PKN model. Both approaches describe one spatial attractor in the center of the medium.

**Figure 6:**
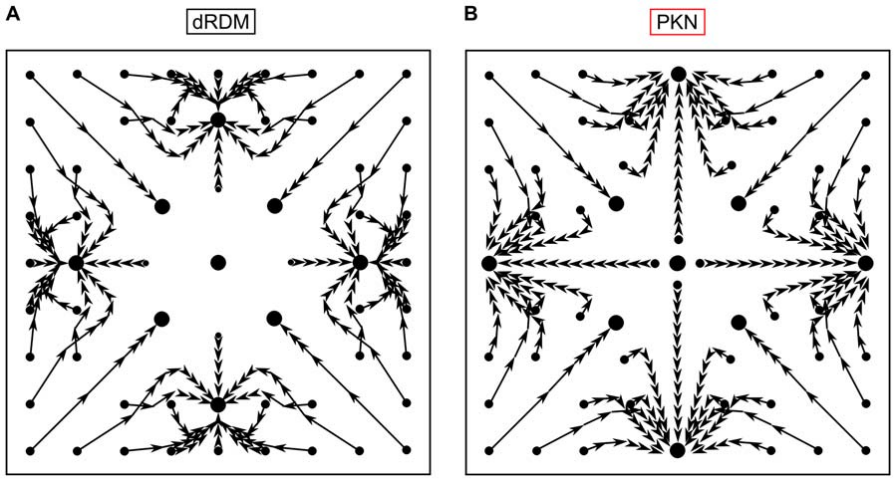
Pacemaker drift in smaller medium (size 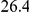 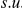). Notations are as in Figure 5. **(A)** dRDM model with , 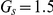 **(B)** PKN model with 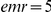, other parameters as in [12].

Figure 6A shows the drift patterns for a smaller system size in the dRDM model with peripheral attractors and attractors on the diagonals of the medium. It should be noted that the diagonal attractors were not previously reported in [12]. However, we performed the same experiment using the PKN model (Figure 6B) and found that these attractors indeed existed using the continuous PKN approach. Thus all spatial attractors were present in both modeling approaches.

**Figure 7:**
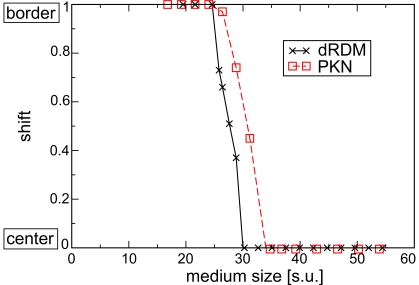
Spatial location of the attractor. Relative shift (location of the peripheral attractors as a proportion of the distance from the center to the boundary of the medium) against medium size. 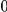 corresponds to the attractor located at the center of the medium and 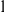 to the attractor located at the border of the medium. Computations with the dRDM model (black symbols, continuous black lines) were performed using 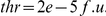, 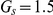, 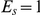 and 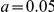. The results from the PKN model (red squares, dotted red line) are from [12].

We also studied how the location of the peripheral attractors depended on the medium size. Figure 7 demonstrates the distance of the peripheral attractor from the center on a graph similar to that in [12]. Although the elastic properties of PKN and the dRDM model are not identical, the drift patterns showed qualitative agreement. Both modeling approaches demonstrated that there is a shift of peripheral pacemaker attractor locations to the center of the medium as the size of the model is increased. Additionally, this transition occurs at comparable sizes of the medium: 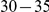 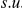. Therefore, we conclude that the dRDM model reproduces the same phenomena on pacemaker activity as reported in [12].

The increased resolution of the dRDM model (compared to [12]) allows one now to study this system in greater detail. In particular, we shift the focus now onto the following open issues: the effects of change of medium size on the pacemaker period; and the mechanisms underpinning pacemaker drift.

#### Pacemaker period

**Figure 8:**
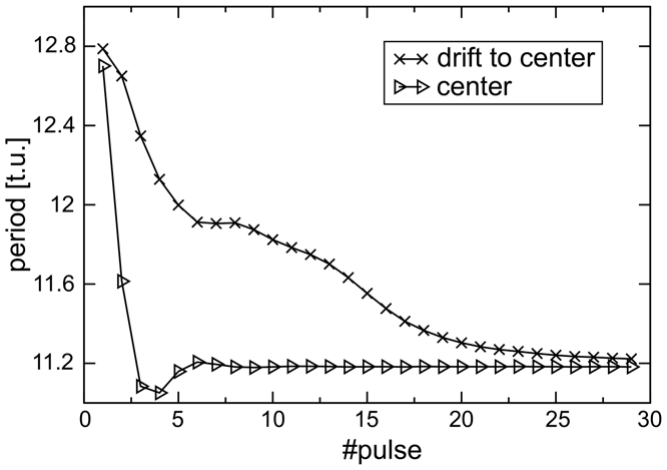
Spatio-temporal study of pacemaker period. Pacemaker period for a pacemaker drifting from the boundary of the medium (initiated 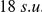 from the center for the medium size of 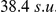) to the center, in comparison to the period of a pacemaker that was initiated at the center.

This section is devoted to the cases of pacemaking activity that result in a static pacemaker located at the center of the medium. The aim of this section is to understand the factors that determine the period of the pacemaker and its dependency on the medium size. This investigation commenced with the study of the spatial and temporal transient processes leading to the steady state configuration of a pacemaker with a constant period located at the center of the medium. Figure 8 illustrates how the period of a pacemaker of the large system shown in Figure 5 evolves during the drift of the pacemaker to the center of the medium. The results of two simulations are shown: for a pacemaker that was initiated at the center of the medium (the red line) and for a pacemaker that was initiated at the boundary of the medium (the black line). In both cases, the pacemakers initially had a long period that rapidly decreased over 3–5 cycles. Following this transition phase, the period of the centrally located pacemaker rapidly settled to the value of 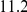 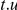. For the peripherally located pacemaker, its period rapidly decreased during the transition phase to 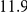 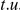 and then the period slowly decreased further during the drift process. By the time the pacemaker had reached the center of the medium, its period had approached the same value of 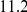 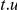. Therefore, the drift of a pacemaker to the center can be described as drift to a region of shorter period.

**Figure 9:**
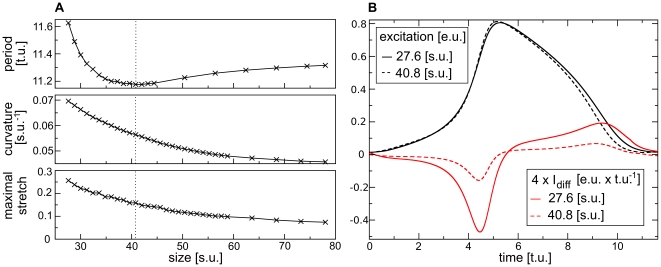
Pacemaker period. **(A)** The ‘stretch regime’ and the ‘curvature regime’ of the pacemaker period (separated by a dotted line in each panel). (Upper panel) Pacemaker period *vs* medium size. (Middle panel) Curvature of a new forming pulse *vs* medium size. (Lower panel) Maximal stretch (scaled using 
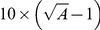
) *vs.* medium size. **(B)** Excitation variable 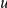 and diffusive current (scaled by 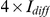) for the center point for one pulse of a pacemaker in a system of size 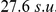 and a system of size 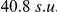.

The results on the study of how the medium size affects the equilibrium period of a stationary pacemaker located in the center of the medium are shown in Figure 9A (upper panel). Biphasic behavior was observed. For system sizes larger than 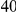 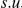, the equilibrium period decreased with a decrease in the medium size. On the other hand, for system sizes smaller than 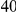 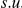, the steady-state period increased with a decrease in the medium size. This biphasic behavior is explained in the following. The first regime is the result of an increase in the maximal stretch of the medium. Figure 9A (lower panel) shows that the maximal stretch monotonically increased with a decreasing medium size. This observation was qualitatively reported in a previous study using the continuous PKN description [12]. The larger stretch resulted in a larger stretch-activated current 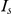, which in turn resulted in a shorter period. The second regime occurred due to a different mechanism. The decrease in medium size also resulted in a decrease of the size of the pacemaker. Figure 9A (middle panel) shows the monotonic increase of the curvature of a new forming pulse of a pacemaker with decreasing medium size. This resulted in an increasing influence of the curvature on wave propagation.

Curvature effects are well known in the theory of excitable media [35] and can be explained using the following formal consideration. If a polar coordinate system (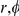) is used to describe the dynamics of a radially expanding wave front, then the expression for the Laplacian will be given by:
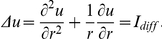



For an expanding wave front 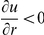, and thus the curvature related term 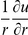 results in a negative diffusive current. This negative diffusive current reduces the velocity of wave propagation and for higher curvature results in the critical curvature phenomenon, i.e. the inability of the wave front to propagate, if its curvature exceeds a critical value. However, for the wave back 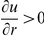, which results in a positive diffusive current that tends to prolong the action potential. Both of these effects are important to understand the second branch of Figure 9A (upper panel). Indeed, comparing the shapes of action potentials for medium sizes 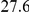 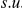 and 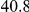 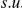 as shown in Figure 9B (black lines), one sees that the upstroke of the action potential was slightly slower in the smaller medium compared to the larger medium (due to negative curvature related current). The recovery process in the smaller medium was also slower (due to the curvature effect on the wave back). This is also illustrated in Figure 9B via the diffusive current (red lines), which showed a larger amplitude for the smaller medium that in turn slowed down the upstroke and prolonged the action potential duration. This prolongation increased the period of a pacemaker (see Figure 9A). When the medium size was decreased below 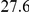 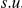, the firing area became smaller than the critical size and the pacemaker activity disappeared. Indeed, for the medium described with the dRDM model (without deformation) the critical curvature found was 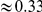 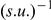, which is close to the curvature 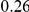 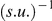 below which a block of the pacemaking activity was observed. Therefore, one can conclude that there are two regimes of dependency of the period of a pacemaker on medium size: the ‘stretch regime’, where the decrease of the period for a decreasing medium size is a result of the increase in maximal stretch; and the ‘curvature regime’, where for a decreasing medium size the period increases and finally the pacemaking activity is blocked due to curvature effects.

#### Mechanisms of pacemaker drift

**Figure 10:**
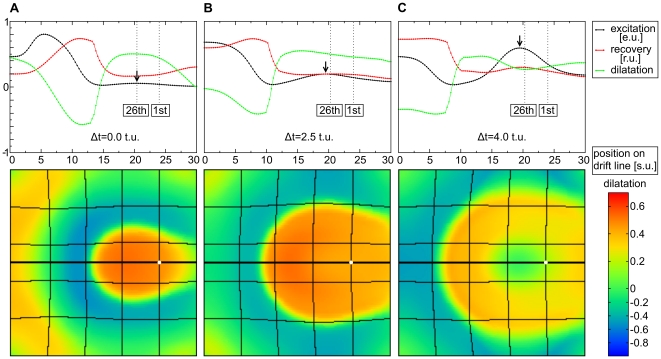
Mechanism of pacemaker drift towards center of the medium. **(A)** emergence of the 26th pulse (at 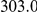 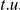). **(B)** The same pulse after 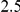 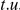 (at 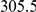 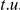) and **(C)** after 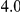 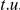 of its emergence (at 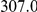 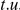). The traces in the upper panels illustrate main state variables in the medium along the pacemaker drift lines (thick black horizontal lines in the lower panels): the excitation variable (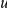) (black), recovery state (scaled using 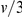) (red), and regional dilatation (scaled using 
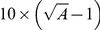
) (green). The locations of the emergence of the 1st and 26th pulse are marked by vertical dotted lines in the upper panels. The maximum voltage is marked with an arrow. The lower panel indicates the regional dilatation in the medium (scaled using 
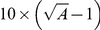
) by a color spectrum. The point of initial stimulation is indicated by a white dot. Medium size 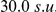.

**Figure 11:**
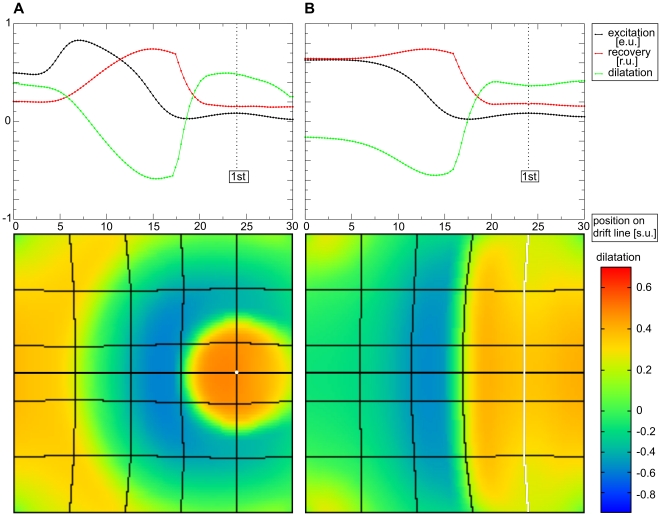
Curved wave front causes stretch asymmetry. Pacemaker stimulation with a point stimulus and a line electrode (shown in white in the lower panels). Snapshots taken at **(A)** 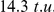, and **(B)** 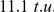. System size and notations are the same as in Figure 10.

This section focusses on pacemaker drift. Figure 10 demonstrates a representative example of pacemaker drift to the center of an RDM medium. It illustrates the formation of the 26th pulse after initiation of pacemaking activity near the boundary of the medium. The lower panel reveals the distribution of local dilatation in the medium and the upper panel illustrates the time course of the main variables of the dRDM model along the pacemaker drift line, which is indicated as a thick black horizontal line in the lower panel. This line indicates the route of the pacemaker during its drift to the center of the medium. The formation of pulse 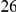 in the tail of the previous (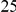
th) wave is shown. The following reasoning is based on the stretch distribution in the medium (the green line) generated by this wave. Initially, the stretch is reasonably symmetric around the new forming pulse (see the green line near the arrow in Figure 10A). However, a clear gradient is evident with higher stretch directed to the center of the medium at a later stage of pulse formation (see the green line near the arrow in Figure 10B). As higher stretch produces a higher stretch activated current 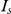, this gradient in stretch leads to a slightly faster depolarization and subsequent excitation closer to the center of the former excitation point (Figure 10C). As a result the subsequent pacemaker position is shifted towards the center of the medium and so on until the pacemaker ended up at the center of the medium. From this, one can conclude that the main driving force of the drift in this case is the asymmetry of the stretch pattern. But why does this asymmetry occur? To study the influence of curvature on the stretch distribution and the pacemaker drift, we compared two cases: a pacemaker initiated by a point stimulus; and a pacemaker initiated by a line electrode. Figure 11 shows the stretch distribution and main variables along the drift line immediately prior to the first pacemaker pulse following the stimulus. Again, a gradient in stretch was evident following the point stimulus (Figure 11A). However, this asymmetry was not present for the line stimulus (Figure 11B). This indicates, that indeed the curvature of the wavefront causes the spatial asymmetry in stretch. Yet, we could not further study a ‘line-shaped’ pacemaker, because after a transient process the initial line-excitation pattern fused to an excitation pattern similar to that following the point stimulus.

**Figure 12:**
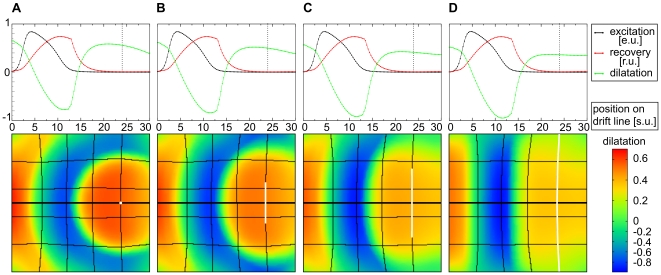
Stretch gradient as function of wave shape. Waves of different curvature generated by electrodes of different shape (shown in white in the lower panels). Snapshots are taken at **(A)** 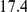 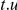, **(B)** 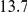 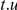, **(C)** 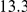 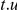, and **(D)** 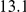 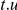. The stretch activated current 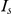 (Eq.(9)) was disabled for these computations. System size and notations are the same as in Figure 10.

To show that the stretch activated current 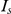 is not important for the formation of the stretch gradient we did similar simulations in the absence of 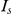. Figure 12A shows the stretch-contraction pattern in this situation. A formation of a gradient in stretch in the vicinity of the previous pacemaker position (around the vertical dotted line in Figure 12A), without stretch activated current is shown. Since stretch is the elastic response to a spatial contraction pattern, we studied of how the shape of the wave front affects the formation of this gradient in stretch. For this study, the wave was initiated by linear electrodes with increasing size, which resulted in the generation of waves with progressively decreased curvature (Figure 12, lower panel). A decrease in curvature decreased the stretch asymmetry until it disappeared for a plane wave stimulus (Figure 12D). Therefore, we conclude that a gradient in stretch in the studied system is formed by the curvature of the wave. Qualitatively this can be understood from the fact that plane wave excitation (contraction) produces stretch mainly in one direction, however, circular contraction ‘pulls’ a point behind a wave front into many directions producing higher maximal stretch than a plane front. This effect is different for points at different distances from the front, which generates a gradient in stretch. A detailed study of the effects of front shape on deformation patterns will be presented as a separate study. The conclusions that can be drawn here is that the drift of a pacemaker to the center in the mechanical setup introduced in [12] is driven by the asymmetry of the stretch pattern, which in turn, is strongly influenced by the shape of the wave front.

As demonstrated in Figure 7, pacemaker drift was directed towards the boundaries of smaller models. There is yet to be a comprehensive understanding of the mechanisms of this drift, but we believe it is related to the ‘curvature regime’ of the period variation as described above. In smaller media the influence of the diffusive current is increased, and it starts affecting the duration of the action potential by inducing a gradient in action potential duration towards the center of the medium. However, to date the authors were unable, to quantify the effects of the diffusive current in relation to drift direction and discriminate them from other observed factors, such as elliptical shape of the firing region, etc. This may serve as a good starting point for follow-up studies.

## Discussion

In this paper, we introduce a discrete modeling framework for the study of reaction-diffusion-mechanics systems. The model is based on the coupling of a mass-lattice model with reaction-diffusion equations. Mass-lattice models are widely used in various areas of computational mechanics research and application [16], [20]. There are several advantages of the dRDM approach presented in this paper. Firstly, its implementation does not require finite element methods, but can be achieved using explicit methods, which allow for a more frequent update rate and higher spatial resolution of the mechanical mesh configuration. Furthermore, the explicit numerical scheme used in this paper to solve the mechanics equations is very effective in studying large systems as the computational speed scales approximately linearly with the number of mass points in the system. The main disadvantage of this approach is that it can not easily be connected to known continuous material properties. However, this does not pose a problem, as we have shown in this paper, that an isotropic Seth material can be used to study basic mechanisms of RDM systems. For more complex materials, it may be necessary to apply homogenization techniques to formulate their constitutive relations. An example of the application of homogenization techniques to derive constitutive relations for a mass-lattice model and its relation to cardiac tissue is given in [36]. It is important to note the possibility to relate discrete mechanics modeling to continuum mechanics by obtaining forces in the mass-lattice model directly from the corresponding constitutive relations [20].

Discrete mechanics modeling and thus our dRDM model is not limited to isotropic material relations. Bourguignon et al. showed that discrete mechanics modeling can be applied to describe elastic properties of anisotropic materials [18]. This approach was further extended to model cardiac elasticity [19], [20]. The extension of the dRDM approach by coupling a RD-model for cardiac excitation to these existing discrete models for anisotropic and hyperelastic cardiac tissue [19], [20] is an interesting approach for the engineering of efficient whole heart models. Furthermore the discrete mechanics description of the dRDM model allows an extension to describe discontinuous deformations [21], [22].

Although cardiac tissue is anisotropic, our isotropic approach can still be applied to several experimental systems. For example the dRDM model is suitable to describe electromechanical processes in cultures of cardiac cells. The tissues produced in these experiments do not show electrical or mechanical anisotropy.

In this work, a discrete mechanical model was coupled with a low-dimensional RD model for cardiac excitation to study the effect of mechano-electrical feedback on cardiac excitation. The phenomenon of pacemaking activity due to stretch activated current was studied, which was previously done using a continuous RDM approach [12]. The dRDM approach not only reproduced all phenomena found with the continuous system, but also allowed us to study them with higher numerical resolution. As a consequence, new properties of pacemakers were identified, such as dependency of the pacemaker period on its location and on medium size. Furthermore, factors that affect the drift of a pacemaker were also identified.

In the continuous modeling approach, the Laplacian in Eq.1 was formulated as a function of the metric tensor to model the influence of deformation on diffusibility [12]. In this paper, however, it is assumed that the main resistance between cardiac cells is not affected during deformation. In the end, this issue was found to be non-essential for the particular problem studied here. Several test simulations using the continuous approach used in [12] were performed and no effects of the different representation of the Laplacian on pacemaker dynamics were found.

## References

[1] Zaikin A, Zhabotinsky A (1970). Concentration wave propagation in two-dimensional liquid-phase self-oscillating system.. Nature.

[2] Winfree A, Strogatz S (1984). Organizing centers for three-dimensional chemical waves.. Nature.

[3] Imbihl R, Ertl G (1995). Oscillatory kinetics in heterogeneous catalysis.. Chem Rev.

[4] Gorelova N, Bures J (1983). Spiral waves of spreading depression in the isolated chicken retina.. J Neurobiol.

[5] Gerisch G (1965). Stadienspezifische aggregationsmuster bei Dictyostelium discoideum.. Wilhelm Roux' Archiv für Entwicklungsmechanik der Organismen.

[6] Weijer C (2004). Dictyostelium morphogenesis.. Curr Opin Genet Dev.

[7] Davidenko J, Pertsov A, Salomonsz R, Baxter W, Jalife J (1992). Stationary and drifting spiral waves of excitation in isolated cardiac muscle.. Nature.

[8] Yoshida R, Takahashi T, Yamaguchi T, Ichijo H (1996). Self-oscillating gel.. J Am Chem Soc.

[9] Bers D (2002). Cardiac excitation-contraction coupling.. Nature.

[10] Kohl P, Hunter P, Noble D (1999). Stretch-induced changes in heart rate and rhythm: Clinical observations, experiments and mathematical models.. Prog Biophys Molec Biol.

[11] Nash M, Panfilov A (2004). Electromechanical model of excitable tissue to study reentrant cardiac arrhythmias.. Prog Biophys Mol Biol.

[12] Panfilov A, Keldermann R, Nash M (2005). Self-organized pacemakers in a coupled reaction and diffusion mechanics system.. Phys Rev Lett.

[13] Panfilov A, Keldermann R, Nash M (2007). Drift and breakup of spiral waves in reaction-diffusion mechanics systems.. Proc Natl Acad Sci USA.

[14] LeGrice I, Hunter P, Smail B (1995). Laminar structure of the heart. II. Mathematical model.. Am J Physiol.

[15] Nash M, Hunter P (2000). Computational mechanics of the heart: from tissue structure to ventricular function.. J Elasticity.

[16] Nealen A, Müller M, Keiser R, Boxermann E, Carlson M (2006). Physically based deformable models in computer graphics.. Computer Graphics Forum.

[17] Delingette H (1998). Towards realistic soft-tissue modelling in medical simulation.. Proceedings of the IEEE.

[18] Bourguignon D, Cani MP (2000). Controlling anisotropy in mass-spring systems.. Eurographics Workshop on Computer Animation and Simulation (EGCAS).

[19] Mohr M, Seemann G, Sachse F, Dössel O (2003). Modeling of myocardial deformation with an extended spring mass system.. Biomedical Engineering.

[20] Fritz T, Jarrousse O, Dössel O, Sloten VanderJ, Verdonck P, Nyssen MJH (2009). Adapting a mass-spring system to energy density function escribing myocardial mechanics.. 4th European Conference of the International Federation for Medical and Biological Engineering.

[21] Popov V, Psakhie SG (2001). Theoretical principles of modelling elastoplastic media by moveable cellular automata method. i: Homogenous media.. Phys Mesomechanics.

[22] Ostermeyer G, Popov V (1999). Many-particle non equilibrium interaction potentials in the mesoparticle method.. Phys Mesomechanics.

[23] Aliev R, Panfilov A (1996). A simple two-variable model of cardiac excitation.. Chaos, Solitons and fractals.

[24] Keener J, Sneyd J (1998). Mathematical physiology..

[25] Feynman R, Leighton R, Sands M (1969). Feynman lectures on physics: mainly electromagnetism and matter..

[26] Schargott M, Popov VL, Heβ M (2007). Macroscopic isotropy of two- and three-dimensional elastic lattice models.. Tribology international.

[27] Krivtsov A (1999). Constitutive equations of the nonlinear crystal lattice.. Z angew Math Mech.

[28] Seth B (1935). Finite strain in elastic problems.. Phil Trans R Soc Lond A.

[29] Hu H, Sachs F (1997). Stretch-activated ion channels in the heart.. J Mol Cell Cardiol.

[30] Zhang Y, Youm J, Sung H, Lee S, Ryu S (2000). Stretch-activated and background nonselective cation channels in rat atrial myocytes.. J Physiol.

[31] Vetter F, McCulloch A (2001). Mechanoelectric feedback in a model of the passively inflated left ventricle.. Ann Biomed Eng.

[32] Trayanova N, Li W, Eason J, Kohl P (2004). Effect of stretch activated channels on defibrillation efficacy.. Heart Rhythm.

[33] Verlet L (1967). Computer “experiments” on classical fluids. i. thermodynamical properties of lennard-jones molecules.. Phys Rev.

[34] Panfilov A (2002). Spiral breakup in an array of coupled cells: the role of the intercellular conductance.. Phys Rev Lett.

[35] Zykov VS (1980). Analytical evaluation of the dependence of the speed of an excitatation wave in two dimensional excitable medium on the curvature of its front.. Biophysics.

[36] Caillerie D, Mourad A, Raoult A (2003). Cell-to-muscle homogenization. application to a constitutive law for the myocardium.. Math Model Numer Anal.

